# Across-frequency combination of interaural time difference in bilateral cochlear implant listeners

**DOI:** 10.3389/fnsys.2014.00022

**Published:** 2014-03-11

**Authors:** Antje Ihlefeld, Alan Kan, Ruth Y. Litovsky

**Affiliations:** ^1^Waisman Center, University of WisconsinMadison, WI, USA; ^2^Center for Neural Science, New York UniversityNew York, NY, USA

**Keywords:** cochlear implant, interaural time difference, envelope ITD, across-frequency integration, spatial sound

## Abstract

The current study examined how cochlear implant (CI) listeners combine temporally interleaved envelope-ITD information across two sites of stimulation. When two cochlear sites jointly transmit ITD information, one possibility is that CI listeners can extract the most reliable ITD cues available. As a result, ITD sensitivity would be sustained or enhanced compared to single-site stimulation. Alternatively, mutual interference across multiple sites of ITD stimulation could worsen dual-site performance compared to listening to the better of two electrode pairs. Two experiments used direct stimulation to examine how CI users can integrate ITDs across two pairs of electrodes. Experiment 1 tested ITD discrimination for two stimulation sites using 100-Hz sinusoidally modulated 1000-pps-carrier pulse trains. Experiment 2 used the same stimuli ramped with 100 ms windows, as a control condition with minimized onset cues. For all stimuli, performance improved monotonically with increasing modulation depth. Results show that when CI listeners are stimulated with electrode pairs at two cochlear sites, sensitivity to ITDs was similar to that seen when only the electrode pair with better sensitivity was activated. None of the listeners showed a decrement in performance from the worse electrode pair. This could be achieved either by listening to the better electrode pair or by truly integrating the information across cochlear sites.

## Introduction

Interaural time differences (ITDs), which occur due to different arrival times of sound energy in the left and right ear, are paramount for localizing the direction of a sound source and for attending to speech in a mixture of surrounding sound (Darwin and Hukin, 1999; Kidd et al., 2005). However, bilateral cochlear implant (CI) listeners do not rely much on ITDs when localizing sound (e.g., Seeber and Fastl, 2008). Moreover, studies on speech intelligibility in situations with background sound demonstrate that binaural processing effects in bilateral CI users are either absent, or much smaller compared to normal-hearing (NH) listeners (Litovsky et al., 2006, 2009; Loizou et al., 2009). This suggests that when compared to NH listeners, CI listeners struggle to effectively utilize ITDs (Ihlefeld and Litovsky, 2012). One of the key challenges for CI listeners is to understand speech in situations with background sound, where ITDs can greatly aid speech understanding in NH listeners. Because speech is a broadband signal, binaural preservation of cues through multi-channel stimulation is ultimately required for restoring speech intelligibility in CI listeners in natural, multi-source environments. A first step toward the goal of restoring multi-channel binaural cues is to examine ITD sensitivity in CI listeners when multiple cochlear sites are simultaneously stimulated, and this is the focus of the current study.

Most CI listeners can resolve ITDs in the signal envelopes, at least for 100% modulated pulse trains in quiet, but are insensitive to fine structure ITDs transmitted by high carrier rates (Lawson et al., 1998; van Hoesel and Tyler, 2003; Laback et al., 2004). Previous work shows that CI listeners can discriminate envelope-ITDs with thresholds as small as 50 μ s (van Hoesel et al., 2009), with an overall sensitivity that shows U-shaped tuning as a function of envelope modulation frequency and peak sensitivity around 100 Hz (Noel and Eddington, 2013). However, when stimulation rate is low, there is generally reduced potency of fine structure ITD cues in bilateral CI listeners, and it is common to observe idiosyncratic differences in ITD sensitivity across cochlear sites (Litovsky et al., 2012). One reason is that within each ear, electric fields from stimulating electrodes can spread to nearby sites, resulting in reduced sensitivity to stimulation at desired electrodes (e.g., Bierer, 2010). Moreover, within and across listeners, there are interaural differences in neural survival and electrode placement which leads to reduced ITD sensitivity (Kan et al., 2013). Differences in envelope ITD sensitivity across stimulation sites could affect how CI listeners interpret broadband envelope ITD information.

In addition to these idiosyncratic factors, the acoustic environment could also influence across-electrode integration of envelope ITD. From an acoustic perspective, usefulness of envelope-ITDs is known to have limits. ITD robustness can be much reduced in everyday environments, where sound energy reflected from walls and ceiling, and energy from competing acoustic sources superpose with target sound. Reverberation often reduces the modulation depth of a target source relative to quiet anechoic conditions, decreasing both speech identification and sound localization performance (Houtgast and Steeneken, 1984; Watkins, 2005; Ihlefeld and Shinn-Cunningham, 2011; Ruggles and Shinn-Cunningham, 2011). Under anechoic conditions, potency of envelope-ITDs depends on the rise time of the slope of the envelope and on the duration of gaps in the envelope, both of which generally vary with modulation depth (Klein-Hennig et al., 2011; Laback et al., 2011; Dietz et al., 2013). This raises the question of how the auditory system can accommodate interference from reduced modulation depth in CI listeners. The effects of demodulation may be even more detrimental on performance for CI than for NH listeners. Indeed, when bilateral CI listeners localize sounds in a noisy background, the signal to noise ratio at which they can effectively utilize envelope-ITDs is much higher than for NH listeners (for a recent review, see van Hoesel, 2012).

Few previous studies have systematically investigated the role of modulation depth for ITD sensitivity in CI listeners. Early work on bilateral CI listening reported results for single-site stimulation in one CI listener, whose ITD sensitivity improved with increasing modulation depth (van Hoesel and Tyler, 2003). More recent work on CI listeners shows that, for trapezoidally amplitude modulated 1515 pps carriers, envelope-ITD sensitivity improves with decreasing duty cycle of the envelope (Laback et al., 2011). CI simulations with NH listeners confirm this effect of duty cycle and further suggest that envelope-ITD sensitivity improves monotonically at low modulation depths, then saturates beyond a critical modulation depth (Bernstein and Trahiotis, 2011; Klein-Hennig et al., 2011; Laback et al., 2011; Dietz et al., 2013).

It is notable that previous work on CI envelope-ITDs has focused on single-site stimulation (van Hoesel and Tyler, 2003; Laback et al., 2011; Noel and Eddington, 2013). However, most natural sounds have broadband energy. For CI stimulation it is therefore desirable to provide envelope-ITDs concurrently at multiple sites along the cochlear array. The current study examines broadband ITD sensitivity in bilateral CI listening, when stimulation occurs at two cochlear sites, and when ITDs are consistent across sites. The aim was to examine the integration of envelope-ITD cues that are spectrally remote due to their positioning at two anatomically different places along the cochlear arrays. Two experiments compared envelope-ITD sensitivity in CI listeners for stimulation that was at a single site vs. dual-site stimulation.

Previous work showed that some CI listeners depend heavily on onset ITD cues (Laback et al., 2007; van Hoesel, 2008). Unlike ongoing cues, due to their transient nature, onset cues do not offer multiple independent “looks” and thus do not provide the opportunity to combine sensory information across time. For an optimal listener, the availability of ongoing cues should improve performance relative to having only onset cues (Hafter and Dye, 1983). To gauge the relative importance of onset cues on integrating binaural cues across electrodes, Experiment 1 measured performance with onset cues; Experiment 2 tested four listeners with “good” binaural sensitivity as determined by data from Experiment 1, with attenuated onset cues.

Of interest was how bilateral CI users who are stimulated at two sites along the cochlear array extract binaural cues, in particular if, due to neural-electrode interface or neural survival issue, these listeners have different sensitivity to binaural cues at the two sites. In one potential scenario, these listeners might only process the binaural cue that is most salient. Thus, CI listeners may interpret binaural cues from the two cochlear sites as one aggregate spatial percept, and their performance with two binaural pairs of electrodes is not expected to increase relative to the single-site stimulation. We also consider the possibility that dual-site stimulation may increase a listener's uncertainty as to what cues to listen to, causing mutual interference and reducing ITD saliency. In this second scenario, ITD sensitivity would decrease in the aggregate sound compared to listening to the better single-site alone. Another possible outcome would be that of enhanced performance with dual-site vs. single-site electrodes; this might occur if CI listeners can combine ITD cues from multiple pairs of electrodes that are treated as independent channels of information.

Here we provide behavioral evidence buttressing the early finding that envelope-ITD sensitivity improves with increasing modulation depth (van Hoesel and Tyler, 2003). Furthermore, we show that when two cochlear sites jointly convey ITD information, CI listeners perform no worse than when they listen to the better single site. Six out of eight tested bilateral CI listeners either showed an improvement in ITD *d*′-sensitivity when two cochlear regions were stimulated jointly, or their performance was similar to that with the better of the two electrode pairs. None of these six listeners showed consistent interference from the worse electrode pair. Two additional performers, with very poor ITD sensitivity, showed neither consistent improvement nor decrement in performance for dual- vs. single-site stimulation.

## Materials and methods

### Listeners

Eight bilateral CI users with Nucleus devices participated in the study and were paid for their time. All testing was administered according to the guidelines of the Institutional Review Board of the University of Wisconsin-Madison. Table 1 lists details of their clinical etiology.

**Table 1:** **Clinical etiology, device: nucleus 24-electrodes**.

**CI listener**	**IAZ**	**IBB**	**IBF**	**IBK**	**IBP**	**IBU**	**IAJ**	**IBR**
Etiology	Unknown	Progressive sensori-neural	Hereditory	Hereditory	Hereditory	Bacterial meningitis	Unknown	Progressive sensori-neural
Age (years)	77	45	62	57	70	64	65	56
Use duration (months)	R(49)	R(57)	R(63)	R(48)	R(25)	R(86)	R(84)	R(7)
	L(72)	L(36)	L(45)	L(48)	L(96)	L(79)	L(168)	L(4)
Stimulation rate per channel (Hz)	1200	1800	900	1800	1800	900	1200	900

### Stimuli

Custom-written software (implemented in Matlab, The Mathworks, Natick, MA) was used to present the stimuli and record listeners' responses. Stimuli were delivered with two synchronized Nucleus Implant Communicators (Cochlear Ltd., Sydney, NSW, Australia). Prior to testing human listeners, we confirmed the proper function of the custom-written stimulation software, by projecting the output from two test implants to an oscilloscope across a range of ITDs. Moreover, as a precaution at the beginning of each testing day, we checked proper function of our equipment with an oscilloscope.

**Figure 1:**
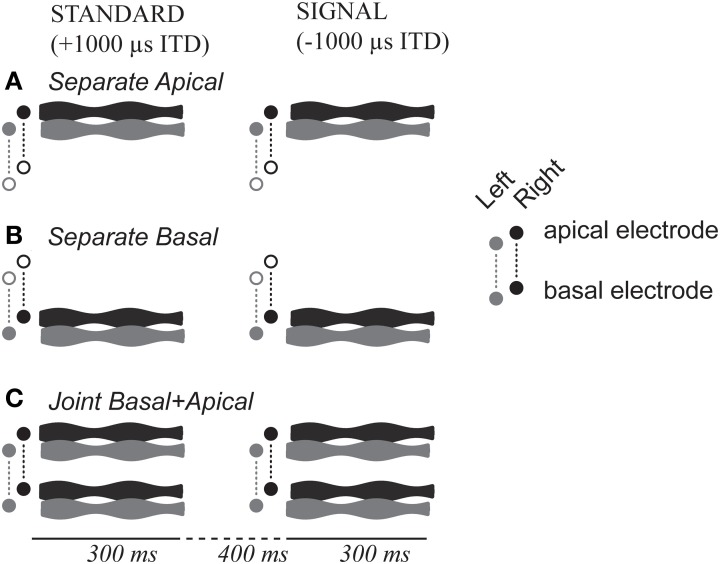
**Task and stimulus design**. Stimuli could be presented via either **(A)** basal-only, **(B)** apical only, or **(C)** dual-site electrode configuration. The two cochlear sites are shown in the vertical dimension of each panel, left and right ear are shown in gray and black, time is shown along the horizontal dimension.

Each stimulus consisted of a 300-ms long, 1000 pps pulse train that was 100-Hz sinusoidally amplitude-modulated (Figure 1). Electrodes were activated in monopolar configuration. Pulses were biphasic, with phase duration of 25 or 50 μ s, depending on the comfortable loudness range of each CI listener (cf. Table 2). Envelopes (*E*) of each amplitude modulated pulse train were as follows:

(1) $$E(t)=I_{\text{max}}-m/200*(I_{\text{max}}-I_{\text{min}})*[1+\cos(2*pi*f*t)],$$

where *I*_max_ and *I*_min_ are the maximal and minimal amplitudes in clinical units, respectively, *m* denotes the modulation depth in percent, *f* equals 100 Hz, and *t* denotes time. *I*_min_ equaled detection threshold (T-level) for a 1000 pps pulse train with 0% modulation depth. *I*_max_ was set such that, for single-site stimulation, the overall stimulus loudness was the same across modulation depths (see Loudness Calibration Procedures below). Throughout testing, *I*_max_ was held fixed for each electrode and modulation depth. As a result, component stimulation levels in the dual-site stimulus were identical to the single-site cases. The starting phase of the envelope equaled zero.

**Table 2:** **The left and right (L/R) electrodes in each pair, the threshold level I_min_ in clinical units (cu), and the most comfortable level I_max_, for 0% modulation depth at 1000 pps**.

**CI listener**	**IAZ**	**IBB**	**IBF**	**IBK**	**IBP**	**IBU**	**IAJ**	**IBR**
Basal	8/8	8/8	6/7	6/8	4/6	9/6	6/9	4/5
*I*_min_ [cu]	117/127	111/133	123/113	148/115	140/145	118/113	147/172	125/112
*I*_max_ [cu]	174/150	178/178	176/169	215/185	173/169	147/157	176/195	149/154
Apical	18/18	20/19	18/18	16/18	20/21	20/18	19/20	18/20
*I*_min_ [cu]	120/128	110/138	132/129	165/132	142/106	130/111	123/171	110/130
*I*_max_ [cu]	156/158	169/185	179/180	225/183	168/153	150/155	139/201	168/172

1000 pps pulse trains were multiplied with the envelope and waveforms were time-delayed across ears, generating 1000 or 2000 μ s ITDs. We initially presented 1000 μ s ITD, expecting that it would lead to perfect performance at 100% modulation depth (van Hoesel et al., 2009). However, some listeners were unable to do the task with 1000 μ s ITD during initial training. Those listeners were tested with 2000 μ s ITD instead.

### Calibration procedures

Three types of calibration procedure were performed. First, a bilateral pitch-matching task was conducted to select electrodes whose combined stimulation produced binaurally fused percepts (see Methods in Litovsky et al., 2010). Second, a loudness-balancing task was performed to ensure that all single-site stimuli were presented at a similar overall loudness. Third, an interaural level difference (ILD) centering was conducted so that the sounds were perceived roughly in the center of the head for an ITD of zero.

#### Pitch matching

For bilateral pitch matching, we initially selected two basal and two apical electrodes in the left ear. For each of these left-ear electrodes, we selected five right-ear electrodes whose clinical frequency stimulation ranges were close to that of the left ear. With a 1000 pps unmodulated pulse train stimulus, we sequentially stimulated the left ear electrode followed by one of its potential pitch matched electrodes in the right ear and asked the listener to categorize the perceived pitch of the second stimulus by determining whether, relative to the first stimulus, it was heard as: much lower, slightly lower, similar, slightly higher, or much higher. Twenty trials were collected per electrode pair for each listener. To reduce the possibility of range effects, all potential apical and basal pairs were presented in random order. This pitch comparison task resulted in one basal and one apical bilateral pair of electrodes that listeners most consistently rated similar in perceived pitch across ears. Table 2 lists the electrode numbers for each listener comprising left-right pairs.

#### Loudness balancing

Each listener performed a series of loudness balancing calibrations (Landsberger and McKay, 2005). For each loudness-balancing track, two sounds, a fixed loudness reference and a level-adjustable target, were presented sequentially in two stimulus intervals. The level-adjustable target was initially set to a quiet level. The listener controlled the stimulus and increased the target level *I*_max, Target_ until the target sounded louder than the reference, then decreased the target level until the target sounded softer than the reference, followed by another increase in level until both reference and target sounded equally loud. Two tracks were recorded, and subsequently the roles of the two sounds were reversed and testing was repeated for two additional tracks. The signed difference between the target and reference, averaged across all four tracks was then added to the initial reference level, resulting in the loudness-balanced target level.

This loudness balancing was initially performed with 1000 pps unmodulated pulse trains, once across the two left-ear electrodes, and once across the two right-ear electrodes. The resulting sounds were near most comfortable level (C-level) on all four electrodes. In each ear, the basal and apical electrodes were then presented jointly at current levels that were a few dB below these loudness balanced current levels, and their levels were gradually increased together until they sounded comfortably loud when presented jointly. Specifically, for the dB scaling, the output level in current level units (CLU) was converted into units of Ampere, scaled in dB and then converted back in to CLU.

#### ILD centering

Loudness balancing tracks were followed by ILD centering where all four electrodes were stimulated simultaneously, and the listener was asked to adjust the perceived intracranial location of the sound until it was perceived to be in the center of the head. The adjustment was made by lowering the stimulus level on the side that dominated perceived laterality of the sound image.

After ILD centering, another round of loudness balancing measurements followed. For each of the four electrodes, the modulated sound was adjusted with the loudness balancing routine described above, balancing unmodulated 1000 pps with each of 100, 20, and 60% modulation depth (in that order). Specifically, the reference sound was the unmodulated 1000 pps train presented at the C-level that would have produced an ILD-centered percept for dual-site stimulation. *I*_min_ was set to the T-level for 1000 pps at that electrode. Modulation depth was held constant. Listeners balanced *I*_max_ of the modulated sound. *I*_max_-values for 10, 40, and 80% modulation depth were then linearly interpolated. As a final verification, we presented all four electrodes jointly at 100% modulation depth, and asked listeners to report the intracranial perceived location. All listeners reported that they perceived a dominant intracranial image approximately near midline. Note that due to monaural loudness summation, the dual-site, ILD-centered C-levels were smaller or equal to the C-levels for electrodes stimulated in isolation. Moreover, some listeners showed bilateral asymmetries, whereby they reduced the level of their better ear well below that ear's isolated C-level in order to obtain a centered intracranial image.

### Testing procedures

Pitch-matched, loudness-balanced, ILD-centered stimuli were used for ITD discrimination testing. Using a 2I-2AFC task, on each trial, left-leading and right-leading ITDs were presented in random order, separated by a 400 ms inter-stimulus interval (ISI). The listener's task was to identify whether the overall sound image moved from left to right or from right to left across the two stimulus intervals. The order of the intervals with left-leading and right-leading stimulus varied randomly from trial to trial. For training purposes, each listener performed the first two blocks of testing at each electrode condition with correct-response feedback. No feedback was given during the remainder of the experiment.

#### Psychometric functions

For each channel, we measured percent correct as a function of *m*, the envelope's modulation depth. On each trial, *m* was chosen randomly from one of the possible modulation depths. To prevent learning effects, all values of *m* were tested once before being presented again. We initially tested seven modulation depths: 0, 10, 20, 40, 60, 80, and 100%. Occasionally, listeners clearly performed at floor or ceiling levels for some of these modulation depths. For these listeners we focused data collection on the most informative modulation depths. We presented 15 trials per block, and held the electrode configuration and modulation depth constant within each block. Blocks were grouped in triplets. Each triplet consisted of one modulation depth, presented at one apical-only, one basal-only, and one dual-site apical-basal configuration; these configurations were interleaved in a Latin Square Balanced design across blocks. Modulation depth varied randomly across triplet groups and was balanced in Latin Square design. All modulation depths and electrode configurations were tested once before everything was repeated with new randomization. There were four overall repeats of all testing conditions, resulting in 60 trials per electrode configuration and modulation depth and CI listener. All listeners completed the testing within 1 day. During training, even in the easiest, 100% modulated condition, two listeners (IAJ, and IBR) struggled to discriminate between movement from +1000 μ s ITD to −1000 μ s ITD vs. the opposite direction. Those listeners were tested with ±2000 μ s ITD instead.

Time permitting, listeners who could perform the ITD discrimination task with ±1000 μ s ITD participated in a second ITD discrimination experiment with ramped onsets and offsets. Compared to Experiment 1, the only difference in Experiment 2 was that the stimulus onsets and offsets were ramped with raised-cosine ramps of 100 ms rise and 100 ms fall time. Four listeners completed Experiment 2.

### Data analysis

For each listener and experimental condition, percent correct scores were calculated and converted into *d*′-values. Probabilities of hits for each stimulus interval, *P*_1_ and *P*_2_, were estimated and, to prevent numeric instabilities, bracketed within the range of 1/*N* and 1 − 1/*N*, where *N* equals 60, the number of trials. Response biases were removed and unbiased *d*′-values were calculated by averaging the normal deviates of these probabilities (Klein, 2001):

(2) $$d^{\prime }=\sqrt{2}*\left[z\left(P_{1}\right)+z\left(P_{2}\right)\right]/2.$$

For an optimal listener, with independent internal peripheral noises and no central decision making noise, predicted performance in the dual-site conditions equals

(3) $${d^{\prime }}_{\text{pred}}=\;\sqrt{{d^{\prime }}_{b}{}^{2}+{d^{\prime }}_{a}{}^{2}}$$

where *d*′_*b*_ and *d*′_*a*_ are the *d*′ sensitivities in the basal and apical single-site conditions (e.g., Gockel et al., 2010).

## Results

### Psychometric functions

**Figure 2:**
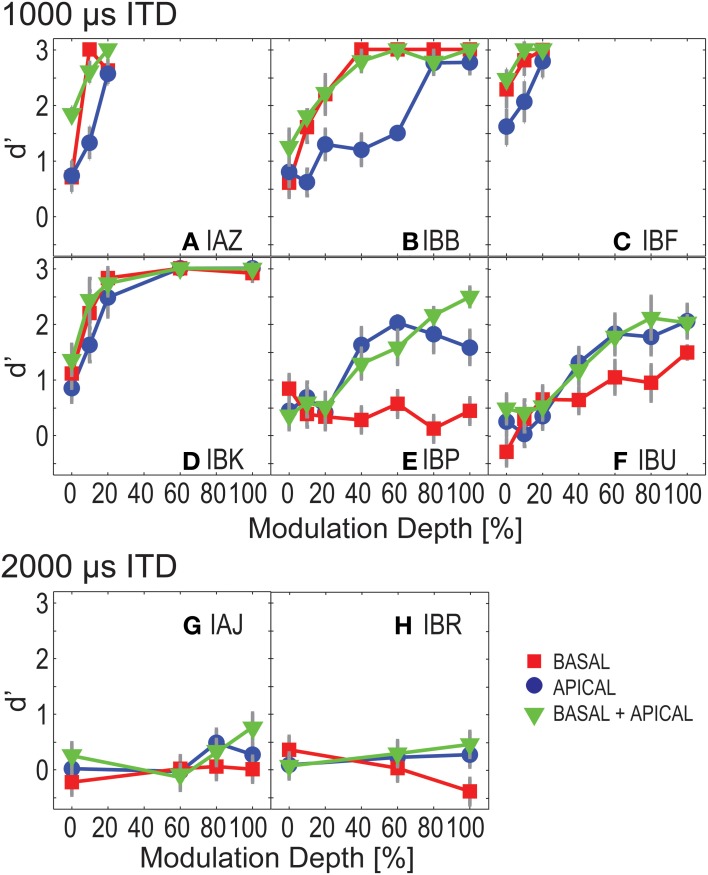
**ITD sensitivity as a function of modulation depth for Experiment 1**. Each panel shows results for one listener. Basal-only, apical-only, and dual-site electrode configurations are shown by the different symbols. Panels show performance of “good” listeners **(A–F)** and poorer performers **(G–H)**.

Figure 2 shows ITD discrimination performance as a function of modulation depth for each of the eight listeners. Panels **A–F** show *d*′ performance for those listeners who could discriminate ±1000 μ s ITD, whereas panels **G,H** show data from the two listeners who could not discriminate ±1000 μ s and were therefore tested with ITDs of ±2000 μ s. Basal-only, apical-only, and dual-site electrode conditions are denoted by square, circle, and triangle symbols. Error bars, where large enough to be visible, show one standard error of the individual *d*′, assuming binomially distributed response rates.

For the six listeners with “good” sensitivity to ITDs (Figures 2A–F), performance generally improved with increasing modulation depth. An exception is IBP whose performance was near chance for the basal electrodes. Four of these listeners performed better for basal than for apical electrodes (squares fall above circles for IAZ, IBB, IBF, and IBK), while the other two listeners performed better at the apical electrodes (IBP and IBU).

None of the listeners performed consistently worse in the dual-site than in the single-site conditions (in Figure 2, triangles generally fall above or coincide with squares and circles). Occasionally, performance in the dual-site conditions fell slightly below that of the better single site. Specifically, for IBB at 40 and 80% modulation depth, and for IBP at 0, 40, and 60% modulation depth, *d*′ sensitivity at the better single-site marginally outperformed dual-site stimulation. However, this was not a consistent trend. Performance was then examined across listeners, to assess whether dual-site stimulation is better than single-site alone. We considered only those modulation depths where all “good” listeners performed the task for all electrode configurations, i.e., modulation depths smaller than or equal to 20%. Repeated measures ANOVA resulted in significant effects of electrode configuration and modulation depth [*F*_(2, 10)_ = 11.487 and 9.128, *p* = 0.003 and 0.025 with Greenhouse–Geisser correction, for electrode configuration and modulation depth].

*Post-hoc* least significant difference (LSD) testing revealed significant differences between all pairwise comparisons of electrode configurations (*p* = 0.026, 0.025, 0.016 for basal vs. apical, basal vs. dual-site, and apical vs. dual-site). Specifically, performance in the basal condition was slightly better than in the apical condition (by an estimated marginal mean difference of 0.4 *d*′-units), indicating that the nominal high-frequency places of stimulation were more sensitive to ITD than the low-frequency places. Moreover, performance in the dual-site condition was slightly and consistently better than in the basal- and apical-only conditions (by estimated marginal mean differences of 0.2 and 0.5 *d*′-units). This suggests that as a group in the dual-site configuration listeners were not simply basing their decision on the apical pair or the basal pair.

The minimal required modulation depth to reach ceiling performance varied strongly across listeners, with IAZ and IBF performing at *d*′ = 3 with as shallow as 20% modulation depth, and IBP and IBU not approaching ceiling performance until 100% modulation depth.

A noteworthy caveat, overall dynamic ranges varied across listeners, perhaps contributing some of the observed variance in the ITD discrimination performance. To address this potential confound, all dual-site-configuration curves were fitted with probit functions. To that end, the *d*′-values in Equation (1) were inverse transformed, and the resulting unbiased percent correct scores were then fit with probit functions. For each listener and electrode, dynamic ranges were calculated as the dB difference between *I*_min_ and *I*_max_ current amplitudes at 100% modulation depth. The slopes in the dual-site electrode conditions vs. each listener's smallest dynamic range in dB were not significantly correlated (*R*^2^ = 0.01, *p* = 0.99). Probit mean and dynamic range were also not significantly correlated (*R*^2^ = 0.56, *p* = 0.24). This suggests that overall dynamic range did not dramatically affect ITD performance.

### Considering the optimal listener model

When apical and basal electrodes were presented jointly, all listeners tended to perform similarly to what would be expected based on optimal integration of the ITD information across the two electrode pairs [Equation (2)]; however, subsequent statistical analysis failed to differentiate between true integration and listening to stimuli presented only to the site with better ITD sensitivity.

Pooled across all listeners and the three lowest modulation depths (0, 10, and 20%), theoretically optimal *d*′ and observed dual-site *d*′ were highly correlated (*R*^2^ = 0.931, *p* < 0.0001). Moreover, when contrasting predicted and observed performance in the dual-site conditions, repeated measures ANOVA found no significant differences between predicted values and observed values [*F*_(1, 5)_ = 6.380, *p* = 0.53]; and a significant effect of modulation depth [*F*_(2, 10)_ = 7.758, *p* = 0.009]. However, when comparing better electrode with dual-site performance, those *d*′-values were also highly correlated (*R*^2^ = 0.91, *p* < 0.0001), and repeated measures ANOVA also did not reveal a statistically significant difference between the two conditions [*F*_(1, 5)_ = 0.14; *p* = 0.14, and *p* = 0.01, for better electrode vs. joint performance, and *F*_(2, 10)_ = 7.32, *p* = 0.01 for modulation depth]. Thus, two different interpretations are consistent with these findings. One interpretation suggests that the six “good” listeners were indeed able to optimally combine information across pairs of electrodes. Alternatively, these listeners simply extracted information from the electrode pair with better ITD sensitivity.

### Poorer performers

For the two more poorly performing listeners (subjects IAJ and IBR), *d*′ was overall close to chance (zero), even with ±2000 μ s ITD (Figures 2G,H). At 80 and 100% modulation depth, for the dual-site electrode conditions, subject IAJ's performance was qualitatively better than chance. A paired *t*-test comparing apical, basal, and dual-site performance showed a significant difference between 0 and 100% modulation depth [*t*_(*df* = 2)_ = −4.5, *p* = 0.05]. However, paired *t*-tests did not reveal significant differences between the three electrode configurations [*t*_(*df* = 3)_ = −0.2, *p* = 0.9 for basal vs. dual-site; *t*_(*df* = 3)_ = −1.6, *p* = 0.2 for apical vs. dual-site]. Subject IBR's *d*′-data show that at 80 and 100% modulation depth this listener was somewhat able to discriminate left- and right-leading stimuli. However, *d*′ was negative for the basal conditions, indicating that this listener consistently reported the opposite directions for these sounds. IBR's results did not differ significantly across stimulus conditions [*t*_(*df* = 2)_ = 0.2, *p* = 0.9, paired *t*-test for 0 vs. 100% modulation depth; *t*_(*df* = 2)_ = 0.2, *p* = 0.9 for apical vs. dual-site; *t*_(*df* = 2)_ = 0.4, *p* = 0.8 for basal vs. dual-site]. In summary, the results suggest that of the two more poorly-performing listeners, one was more sensitive to ITDs in the modulated condition than in the unmodulated condition.

### Onset cues

For the four best-performing listeners (IAZ, IBB, IBF, and IBK, Figures 2A–D), even at 0% modulation depth, performance was better than chance; for each of the electrode configurations, *t*-tests for measured *d*′-values vs. *d*′ = 0 revealed statistically significantly results [*t*_(*df* = 5)_ = 2.6, *p* = 0.05 for basal; *t*_(*df* = 5)_ = 4.1, *p* = 0.009 for apical; *t*_(*df* = 5)_ = 4.0, *p* = 0.01 for dual-site]. These results suggest that the four best-performing listeners were able to exploit onset ITD information, consistent with previous findings (Laback et al., 2007; van Hoesel, 2008).

**Figure 3:**
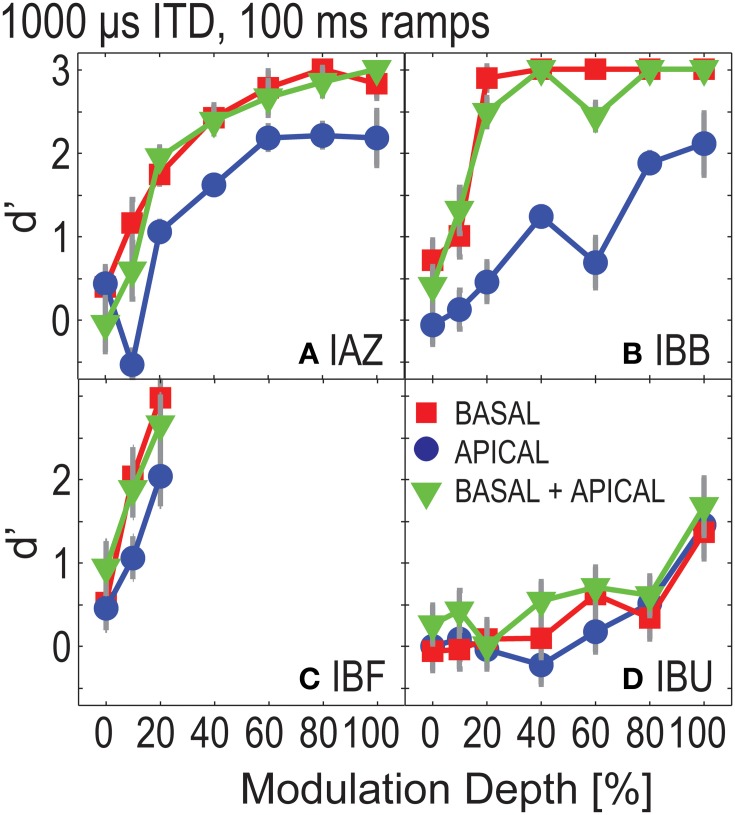
**ITD sensitivity as a function of modulation depth for ramped stimuli in Experiment 2**. Each panel **(A–D)** shows results for one listener. Basal-only, apical-only, and dual-site electrode configurations are shown by the different symbols.

To estimate how well listeners could discriminate ITDs with attenuated onsets, Experiment 2 tested four of the “good” listeners with conditions that were identical to those tested in Experiment 1, except that the stimuli were ramped on and off with 100 ms windows. Figure 3 shows *d*′-performance for the ramped conditions. The overall pattern in performance is similar to that in Figure 2. Performance increases monotonically as a function of modulation depth. Moreover, at each modulation depth, performance in the dual-site electrode configurations is similar to or better than that of the better electrode. Repeated measures ANOVA considering only those modulation depths that had been tested in all listeners and electrode configurations (0, 10, and 20%) found significant effects of modulation depth [*F*_(2, 6)_ = 6.648, *p* = 0.030] and of electrode configuration [*F*_(2, 6)_ = 6.740, *p* = 0.029]. However, *post-hoc* LSD indicated no significant differences between any of the pairwise comparisons across electrode configurations (*p* = 0.091 for basal vs. apical, *p* = 0.692 for basal vs. dual-site, and, *p* = 0.055 for apical vs. dual-site). Pairwise comparisons across modulation depths also found no significant differences with *post-hoc* LSD (*p* = 0.137 for 0–10%; *p* = 0.070 for 0–20%; and *p* = 0.088 for 10–20%).

**Figure 4:**
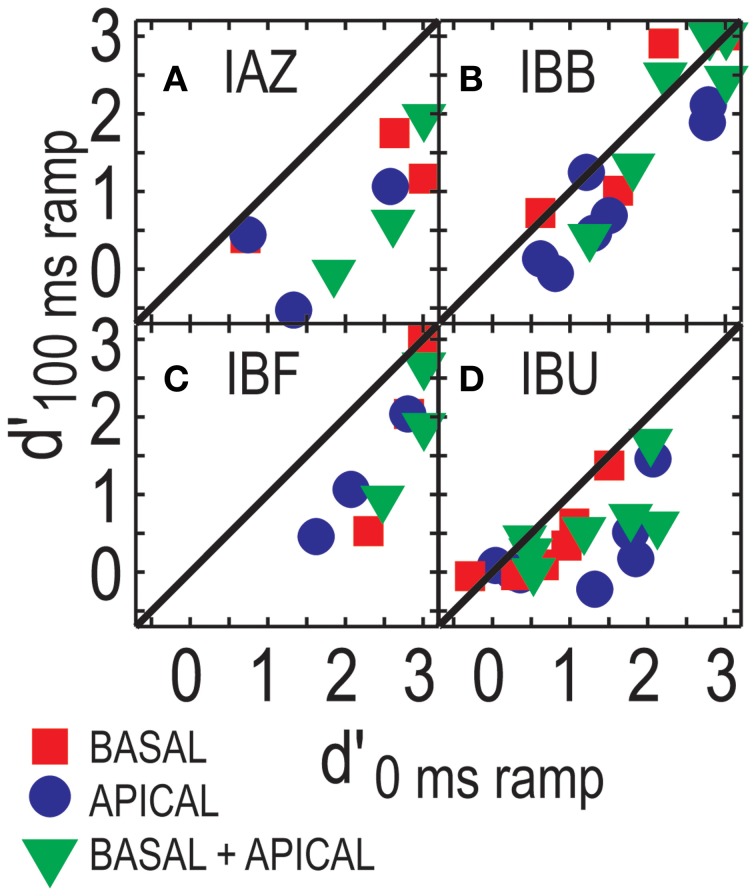
**ITD sensitivity for stimuli with and without ramp, from Experiment 1 and 2, respectively**. Each panel **(A–D)** shows results for one listener. Basal-only, apical-only, and dual-site electrode configurations are shown by the different symbols. Repeated symbols reflect the fact that performance is shown across a range of modulation depths.

Figure 4 shows performance in the ramped conditions as a function of performance in the un-ramped conditions (one data point plotted per modulation depth). Overall, performance in the ramped conditions was worse than that in the un-ramped conditions (points fall below the diagonal). Repeated measures ANOVA, considering only those three modulation depths that were tested for all four listeners in both the ramped and un-ramped conditions, found no significant effect of ramping [*F*_(1, 3)_ = 7.619, *p* = 0.07]. Main factors of electrode configuration and modulation depth remained significant [*F*_(2, 6)_ = 10.783, *p* = 0.01; *F*_(2, 6)_ = 11.596; *p* = 0.009 for electrode configuration and modulation depth]. Note that unlike the data points in Figure 4, this analysis only considers performance above 20% modulation depth, where all listeners were tested. The effect of ramping is non-significant, however, it appears that at least for some listeners ramping can affect performance (e.g., IAZ, where all points fall below the diagonal in Figure 4A).

## Discussion

Natural sounds have broadband energy, giving rise to ITDs along the full spectral range. In order to present binaural cues with fidelity for broadband sounds, it is necessary for CIs to present ITDs concurrently at multiple electrodes that are placed along the cochlear array. Current bilateral CIs present sounds, and therefore ITDs, through envelope information. However, in everyday acoustic settings, sound envelopes are often de-modulated by competing sources and by reverberant energy. In these adverse listening conditions, both CI sound localization and speech intelligibility suffer compared to NH performance (e.g., van Hoesel, 2012). The current study examined how CI listeners were able to combine envelope-ITD information across two concurrent stimulation sites, when modulation depth was parametrically varied. We compared ITD sensitivity across apical and basal cochlear regions when these two regions were stimulated either alone or jointly.

Consistent with early findings for one CI listener (van Hoesel and Tyler, 2003), our results show that ITD sensitivity increased with larger modulation depth for six out of eight CI listeners. Previously, when presented with transposed stimuli, ITD sensitivity in NH listeners decreased and perceived intracranial laterality moved frontally with smaller modulation depth (Bernstein and Trahiotis, 2011). The current results are consistent with the interpretation that, for CI listeners, similar to NH listeners, the intracranial localization strength was more distinctly available with steeper modulations.

There is some disagreement in the literature on CI listeners about whether binaural performance varies with stimulation site (apical or basal). One previous study found no difference across stimulation sites, for high-rate pulse trains (6000 pps) that were sinusoidally modulated at 100 Hz (van Hoesel et al., 2009). The absence of a stimulation site effect is consistent with findings in NH listeners who show similar ITD sensitivity to transposed tones with high frequency carriers and low-frequency pure tones, which stimulate the basal and apical regions of the cochlea, respectively (van de Par and Kohlrausch, 1997). The current study supports those findings. In particular, while each listener had a better electrode pair, there was no consistent across-listener trend that would differentiate apical and basal results: four out of six “good” listeners performed worse at the apical than at the basal pair, for two listeners the reverse was true. Similar findings have been reported for un-modulated low-rate pulsatile stimulation (Litovsky et al., 2010). That study had examined basal, middle, and apical for 100 pps and found no effect of place. However, other data shows that listeners performed better with basal than with apical stimulation, as tested with low-rate pulse trains (100 pps; Best et al., 2011).

Here, basal-alone and apical-alone levels at each electrode pair were usually below C-level for that pair when played in isolation. ITD sensitivity can decrease with decreasing stimulation level (van Hoesel, 2007). Here, we loudness-balanced the stimuli across cochlear stimulation sites. Therefore, it is unlikely that observed differences in ITD sensitivity across basal and apical stimulation sites are an artifact of the stimulus level choices. Instead, differential ITD sensitivity could be due to across-site differences in neural survival and biological or surgical factors, including proximity of electrodes and nerve (Litovsky et al., 2010, 2012). Our results show that in the dual-site electrode configurations, performance was always better than that of the electrode pair producing poorer performance. By loudness-balancing we ensured that performance was not simply dominated by the louder and therefore more salient pair of electrodes.

While previous work demonstrates that interactions across cochlear sites can influence performance for conflicting ITD cues for both acoustic and electric hearing (McFadden and Pasanen, 1975; Best et al., 2011), here, both pairs of electrodes were situated in a more basal position relative to where neurons with best frequencies in the 600–700 Hz range are typically located, and both provided non-conflicting envelope-ITDs, making it unlikely that binaural interference would affect listeners' performance. Indeed, to the extent that binaural interference may have influenced ITD sensitivity, performance in the dual-site conditions should have been worse than that of the single-site basal conditions. No such binaural interference was observed. In fact, the electrode pair with worse performance did not drive performance below that of the electrode pair with good performance in any of the dual-site stimulation conditions, showing that listeners do not suffer from interference even when one electrode pair provides poorly represented spatial information.

An important issue regarding many previous studies with bilaterally implanted CI users is the consideration of subject selection criteria. In a number of prior studies, listeners had been pre-selected based on their abilities to perform a binaural task with low-rate stimuli (e.g., Laback and Majdak, 2008; van Hoesel, 2008; van Hoesel et al., 2009). Here, listeners were not pre-selected according to performance criteria, which may help explain the fact that some listeners could not perform the task with 1000 μ s at 100% modulation depth, even though this has not been reported in the literature before.

A potential confound to note is that modulation depth was measured in percentage of the dynamic range between *I*_min_ and *I*_max_, and this range differed across listeners (Table 2). A listener with a large difference between *I*_min_ and *I*_max_ may have performed better because the sounds were modulated over a wider dynamic range than for a listener with a small dynamic range. However, because the dynamic range was not significantly correlated with task performance, we deem this possibility unlikely.

Some listeners were able to perform the ITD discrimination task even at 0% modulation depth. This is consistent with previous work demonstrating that some listeners can utilize onset cues of high-rate pulse trains to discriminate ITDs, even in the absence of post-onset ITD information (Laback et al., 2007; van Hoesel, 2008; Noel and Eddington, 2013). Similarly, in another previous study using an ITD discrimination task, when tested with 800 and 1200 pps stimuli and 600 μ s ITD, CI listeners could perform the task above chance (Laback and Majdak, 2008). Note that in these prior studies, CI listeners were discriminating between 0 ITD and left or right-leading ITD in a 2AFC paradigm (Laback et al., 2007; Laback and Majdak, 2008; Noel and Eddington, 2013), or they were asked to indicate perceived intracranial position (van Hoesel, 2008). Here, CI listeners judged the direction of a change in ITD of 2000 or 4000 μ s, so the task was considerably easier than in those previous studies.

Experiment 2 tested the usefulness of onset cues by ramping the sounds on and off. Ramping altered the stimuli in two ways. It should have resulted in a more staggered stimulation of the neural population than with the non-ramped stimuli, reducing the potential usefulness of onset cues. In addition, the ramp shortened the perceived duration of the sound. Here, when stimuli were ramped, one listener (IAZ) performed clearly worse than when stimuli were not ramped, whereas other listeners showed less appreciable change in performance. Here, because the ±1000 μ s ITD was an integer multiple of the period of the 1000 pps carrier signal, ongoing temporal fine structure ITDs did not provide unambiguous information about source direction. The relatively robust performance with attenuated onsets is consistent with the previous finding that some listeners can utilize ongoing envelope-ITDs (Noel and Eddington, 2013). Moreover, it suggests that strategies for weighting the onset and running portions of each stimulus differ across listeners. These results are relevant when considering previous work on onset weighting. Using stimuli with a dichotic onset pulse followed by three diotic pulses, Laback et al. (2007) demonstrated that with increasing pulse rate CI listeners tend to rely more strongly on onset cues. Similarly, post-onset pulses exerted stronger effects on CI listeners' ITD sensitivity at 100 pps than they did at 300 and 600 pps (van Hoesel, 2008). These studies share that onset and post-onset pulses carried conflicting ITD information. In contrast, here, all pulses had the same ITD. Therefore, here, post-onset pulses are likely to have helped listeners perform the ITD discrimination task, even when onsets were attenuated.

Recent findings suggest that binaural sensitivity can be poorer when nearby electrodes cause energetic masking (Lu et al., 2010). Moreover, in an ITD discrimination task, the binaural bandwidth eliciting a robust ITD on a target electrode was estimated to be about five times greater in CI than in NH (Poon et al., 2009). Specifically, to decrease ITD sensitivity by a factor of 2 (binaural half-width), the mismatch in cochleotopic position across ears in CI users is 3.7 mm as opposed to 0.7 mm in NH listeners (Poon et al., 2009). This widened binaural bandwidth in CI users as compared to NH listeners could imply higher susceptibility to energetic masking also within one ear. Furthermore, in a binaural masking level difference task, CI listeners' abilities to detect a tone on a target electrode pair improved when fewer electrodes carried the masking signal, even when those additional masking electrodes were outside the nominal critical band for the target electrode (Lu et al., 2010). In addition, the extent of channel interaction, as estimated from auditory nerve evoked potentials in several listeners, was negatively correlated with binaural benefit (Lu et al., 2010). These studies show that peripheral interaction can impact binaural sensitivity, especially when spacing between neighboring electrodes is small. To limit the impact of performance asymmetries across the cochlea due to energetic masking, here, we chose electrodes that were spaced relatively widely. Still, in Experiment 1, each listener had a better electrode pair, similar to previous reports (Laback et al., 2011). Specifically, for three listeners who were tested in both experiments, that better pair was the same across both experiments. Often the differences between apical and basal stimulation within that listener were pronounced with a sizeable difference in midpoints of the underlying psychometric functions. An additional analysis using dB modulation depth along the abscissa (not shown) did not produce qualitatively different trends, nor could we identify a parameter in the level map of the CI listeners that could explain these within-listener differences.

## Summary and conclusions

The current study reinforces and extends findings from previous studies on sensitivity to envelope ITD in bilateral CI listeners. Specifically, when discriminating envelope-ITDs of 100 Hz modulated high-rate pulse trains, a CI listener's performance improves with increasing modulation depth. Moreover, consistent with previous work, most CI listeners performed clearly better at one of the two stimulation sites (Laback et al., 2007; van Hoesel, 2008). However, whether the apical or the basal stimulation site produced higher percent correct scores did not vary consistently across listeners. Finally, some listeners could discriminate 1000 μ s ITDs for an unmodulated 1000 pps train, whereas others struggled with this task. Moreover, when comparing the ramped to the non-ramped conditions, listeners varied in their ability to utilize onset cues. Together, these findings provide further evidence to previous work that listeners differ in how strongly they weigh onset and ongoing cues of the stimulus (Laback et al., 2007; van Hoesel, 2008).

Results show that CI listeners did not perform worse when two electrode pairs jointly transmitted ITD information, compared to listening to the better of the two pairs. This finding is consistent with the interpretation that CI listeners either truly integrated ITD information across the two stimulation sites or that they performed based on the electrode pair carrying more salient ITD information. Performance asymmetries between the two stimulation sites make it difficult to conclusively differentiate between these two explanations. Together these findings provide evidence that CI listeners can sustain ITD sensitivity for two-site stimulation compared to single-site stimulation. The current results are encouraging in that no interference from the worse electrode pair was observed.

### Conflict of interest statement

The authors declare that the research was conducted in the absence of any commercial or financial relationships that could be construed as a potential conflict of interest.
